# Pd_2_Spermine Complex Shows Cancer Selectivity and Efficacy to Inhibit Growth of Triple-Negative Breast Tumors in Mice

**DOI:** 10.3390/biomedicines10020210

**Published:** 2022-01-19

**Authors:** Martin Vojtek, Salomé Gonçalves-Monteiro, Patrícia Šeminská, Katarína Valová, Loreto Bellón, Patrícia Dias-Pereira, Franklim Marques, Maria P. M. Marques, Ana L. M. Batista de Carvalho, Helder Mota-Filipe, Isabel M. P. L. V. O. Ferreira, Carmen Diniz

**Affiliations:** 1 LAQV/REQUIMTE, Laboratory of Pharmacology, Department of Drug Sciences, Faculty of Pharmacy, University of Porto, 4050-313 Porto, Portugal; salomemonteiro8180@gmail.com (S.G.-M.); patka.seminska@gmail.com (P.Š.); katusa.valova@gmail.com (K.V.); loreto.bellon@hotmail.com (L.B.); 2 ICBAS—Instituto de Ciências Biomédicas de Abel Salazar, University of Porto, 4050-313 Porto, Portugal; pdpereira@icbas.up.pt; 3 Clinical Analysis Unit, Faculty of Pharmacy, University of Porto, 4050-313 Porto, Portugal; franklim@ff.up.pt; 4 “Molecular Physical-Chemistry” R&D Unit, Department of Chemistry, University of Coimbra, 3004-535 Coimbra, Portugal; pmc@ci.uc.pt (M.P.M.M.); almbc@uc.pt (A.L.M.B.d.C.); 5 Department of Life Sciences, University of Coimbra, 3000-456 Coimbra, Portugal; 6 iMed.ULisboa, Faculty of Pharmacy, University of Lisbon, 1649-003 Lisbon, Portugal; hfilipe@campus.ul.pt; 7 LAQV/REQUIMTE, Laboratory of Bromatology and Hydrology, Department of Chemical Sciences, Faculty of Pharmacy, University of Porto, 4050-313 Porto, Portugal; isabel.ferreira@ff.up.pt

**Keywords:** Pd(II)-based drugs, cisplatin, metal complexes, triple-negative breast cancer, in vivo, xenografts

## Abstract

Pd_2_Spm is a dinuclear palladium(II)-spermine chelate with promising anticancer properties against triple-negative breast cancer (TNBC), a breast carcinoma subset with poor prognosis and limited treatment options. The present study evaluated the in vitro and in vivo anticancer effects of Pd_2_Spm compared to the reference metal-based drug cisplatin. Triple-negative breast cancer MDA-MB-231 cells, non-cancerous MCF-12A breast cells and chorioallantoic membrane (CAM) assay were used for antiproliferative, antimigratory and antiangiogenic studies. For an in vivo efficacy study, female CBA nude mice with subcutaneously implanted MDA-MB-231 breast tumors were treated with Pd_2_Spm (5 mg/kg/day) or cisplatin (2 mg/kg/day) administered intraperitoneally during 5 consecutive days. Promising selective antiproliferative activity of Pd_2_Spm was observed in MDA-MB-231 cells (IC_50_ values of 7.3–8.3 µM), with at least 10-fold lower activity in MCF-12A cells (IC_50_ values of 89.5–228.9 µM). Pd_2_Spm inhibited the migration of MDA-MB-231 cells, suppressed angiogenesis in CAM and decreased VEGF secretion from MDA-MB-231 cells with similar potency as cisplatin. Pd_2_Spm-treated mice showed a significant reduction in tumor growth progression, and tumors evidenced a reduction in the Ki-67 proliferation index and number of mitotic figures, as well as increased DNA damage, similar to cisplatin-treated animals. Encouragingly, systemic toxicity (hematotoxicity and weight loss) observed in cisplatin-treated animals was not observed in Pd_2_Spm-treated mice. The present study reports, for the first time, promising cancer selectivity, in vivo antitumor activity towards TNBC and a low systemic toxicity of Pd_2_Spm. Thus, this agent may be viewed as a promising Pd(II) drug candidate for the treatment of this type of low-prognosis neoplasia.

## 1. Introduction

Female breast cancer is currently the most commonly diagnosed cancer and the leading type of cancer death in women worldwide, with 2.3 million new cases having been diagnosed in 2020 [1]. Breast cancer is a highly heterogeneous disease that presents several subtypes with different treatment success and survival prognosis. Triple-negative breast cancer (TNBC) stands out as the most aggressive breast cancer subtype, constituting 10–30% of all breast cancer cases. TNBC is characterized by the absence of estrogen receptor and progesterone receptor, as well as HER2 overexpression, and disproportionally affects women with African or Indian ancestry, who show higher incidence of TNBC compared to other breast cancer subtypes [2]. TNBC is also associated with poorer prognosis than other subtypes of breast cancer, mainly due to higher frequency of metastasis, higher relapse rates and limited treatment options, with no conventional receptor-targeting therapeutics available compared to other breast cancer subtypes. However, owing to recent advances in the understanding of the distinctive biology and molecular features of TNBC, the TNBC therapeutic landscape of targeted therapies has been expanded with PARP inhibitors, antibody–drug conjugates and immune checkpoint inhibitors [3]. Nevertheless, platinum(II) chemotherapeutics such as cisplatin and carboplatin still remain the reference treatment of TNBC, either in adjuvant or neoadjuvant settings, and as monotherapy or in combination with other drugs [4,5]. Platinum-based chemotherapeutics are able to increase the disease-free survival time, but the overall survival of patients remains unchanged mainly due to the poor tolerability of the patients (resulting in dose reduction, yielding inferior clinical outcomes) and/or the development of tumor resistance over the treatment time course [4,5]. The most frequent unintended outcomes of platinum-based therapy are related to myelosuppression, gastrointestinal reactions and damage of liver and kidney functions, which may be mitigated to certain level by the pre-hydration of patients and other supporting measures [6].

Palladium-based compounds are being considered as promising analogs to platinum chemotherapeutics, owing to the similarities in structure and coordination chemistry between platinum and palladium ions. In particular, palladium complexes with aliphatic biogenic polyamines have been studied during the last decade, showing promising in vitro antiproliferative activity against several cancer types, including TNBC [7]. A dinuclear palladium complex with spermine (Pd_2_Spm) is a particularly promising agent, with its in vitro antiproliferative activity already reported against various cancer types, including osteosarcoma [8], hormone-dependent breast carcinoma [9], ovarian cancer [10] and TNBC [11]. The pharmacological mechanism of action of Pd_2_Spm is believed to be similar to cisplatin, with both being able to produce drug–DNA crosslinks that lead to cellular death. However, polyamine polynuclear complexes such as Pd_2_Spm have been shown to produce distinctive long-range drug–DNA crosslinks, which are not observed for cisplatin and other classical platinum-based drugs and can prompt more severe and less-repairable DNA damage [12,13,14]. TNBC cells such as MDA-MB-231 cells demonstrated similar antiproliferative effects of Pd_2_Spm and cisplatin [11], but it was reported that breast cancer cells accumulate approximately four times lower amounts of Pd_2_Spm than cisplatin, i.e., a lower intracellular quantity of Pd_2_Spm is required to produce similar antiproliferative effects. This implies the occurrence of alternative (yet unknown) anticancer molecular target(s) for Pd_2_Spm versus cisplatin [15,16,17,18]. A recent pharmacokinetic study in mice also showed that while Pd_2_Spm has a very similar serum terminal half-life to cisplatin, it presents a lower accumulation in major organs as compared to cisplatin [15]. Therefore, Pd_2_Spm is expected to show a superior tolerability as well as lower nephrotoxicity/hepatotoxicity than cisplatin, owing to both lower tissue accumulation and decreased metabolic reactivity with kidney and liver biomolecules responsible for the associated toxicity (which was recently reported by our group) [15,19]. Based on these previous findings, the present study has sought to investigate, for the first time, the in vivo efficacy (to reduce tumor growth) and tolerability (changes in body weight, behavior, histopathological features and biochemical and hematological parameters) of a 5-day Pd_2_Spm treatment compared to cisplatin’s treatment in mice bearing TNBC MDA-MB-231 breast tumors. Additionally, the Pd_2_Spm’s cancer selectivity was also assessed in vitro, along with the effects of Pd_2_Spm on angiogenesis and MDA-MB-231 cell migration, to further broaden our understanding of this chelate. 

## 2. Materials and Methods

### 2.1. Reagents and Chemicals

Cisplatin (*cis*-dichlorodiammine platinum(II), 99.9%), potassium tetrachloropalladate (II) (K_2_PdCl_4_, 98%), spermine (N,N’-bis(3-aminopropyl)-1,4-diaminobutane, 99%), Dulbecco’s Modified Eagle Medium—high-glucose cell growth medium (DMEM-HG), 1:1 mixture of Dulbecco’s Modified Eagle Medium and Ham’s F12 cell growth medium (DMEM/F12 1:1), human epidermal growth factor (hEGF; recombinant, expressed in *E. coli*), cholera toxin from *Vibrio cholerae*, bovine insulin (10 mg/mL insulin in 25 mM HEPES, pH 8.2) and hydrocortisone were purchased from Sigma-Aldrich (Sintra, Portugal). Fetal bovine serum (FBS) and horse serum were from Gibco (Thermo Fisher Scientific, Inc., Waltham, MA, USA). Ultrapure water (18.2 MΩ × cm at 25 °C) was obtained with an arium^®^ pro water purification system (Sartorius, Goettingen, Germany). Animals were anesthetized with isoflurane inhalation (IsoFlo^®^, 100% isoflurane) acquired from Abbott (Berkshire, UK). All the other reagents were of analytical grade. 

### 2.2. Pd_2_Spm Synthesis and Formulation

The Pd_2_Spm complex was synthesized according to published procedures [20] optimized by the authors [21]: 2 mmol of K_2_PdCl_4_ was dissolved in a minimal amount of water and 1 mmol of spermine aqueous solution was added dropwise under continuous stirring. After 24 h, the resulting orange powder was filtered and washed with acetone, and the yellow-orange needle-shaped crystals were recrystallized from water. The composition and purity of the synthesized compound were fully obtained by elemental analysis and vibrational spectroscopy (FTIR, Raman and inelastic neutron scattering), which were compared with the previously calculated vibrational profiles (by ab initio methods) [21,22]. Yield: 68%. Elemental analysis (Pd_2_(C_10_N_4_H_26_)Cl_4_): Found—C: 21.2%; H: 4.7%; N: 9.6%, Cl: 25.9%; Calculated—C: 21.5%; H: 4.6%; N: 9.9%, Cl: 25.6%. A Pd_2_Spm 0.28 mg/mL solution for in vivo administration was freshly prepared by dissolving an appropriate quantity of drug in phosphate-buffered saline (PBS) (H_2_PO_4_ 1.5 mM, Na_2_HPO_4_ 4.3 mM, KCl 2.7 mM, NaCl 150 mM, pH 7.4) containing 0.5% of DMSO and sterile-filtered. A solution of cisplatin 0.35 mg/mL was prepared in PBS and sterile-filtered.

### 2.3. Cell Cultures

The human triple-negative breast cancer cell line MDA-MB-231 (ATCC HTB-26) (absence of estrogen and progesterone receptors, HER2 overexpression) and the non-cancerous breast cell line MCF-12A (ATCC CRL-10782) were purchased from ATCC (Manassas, VA, USA). MDA-MB-231 cells were cultured in DMEM-HG cell growth medium supplemented with 10% (*v*/*v*) FBS. MCF-12A cells were cultured in DMEM/F12 medium supplemented with 20 ng/mL hEGF, 100 ng/mL cholera toxin, 0.01 mg/mL bovine insulin, 500 ng/mL hydrocortisone and 5% (*v*/*v*) horse serum. Cells were cultured in monolayers in a sterile environment at 37 °C with 5% CO_2_ humidified atmosphere. Under these conditions, the population doubling time was 25.5 ± 0.9 h and 20.6 ± 3.1 h for MDA-MB-231 and MCF-12A cells, respectively. The cell cultures were routinely screened for mycoplasma contamination, yielding negative results. 

### 2.4. Cell Proliferation Assay

Cells were seeded in 96-well microplates at the cell density 1.5 × 10^4^ cells/cm^2^ (final volume 200 µL/well) and left 24 h to attach. Afterwards, the growth medium was replaced with the growth medium containing Pd_2_Spm (1–100 µM) or cisplatin (0.1–100 µM). Label-free kinetic live monitoring of cell proliferation was performed using LionheartFX automated microscope (BioTek, Winooski, VT, USA) with direct image acquisition of cells in microplates at 0, 24, 48 and 72 h post-addition of the tested compounds. Acquired 4X images were processed using Gen 5 Image Analysis software (BioTek, Winooski, VT, USA) that allows for identification and counting of individual cells per image. The normalized cell growth (%) was calculated using the following formula:

C(t) = [(Number of Treated Cells(t) − Number of Treated Cells(0))/(Number of Untreated Cells(t) − Number of Untreated Cells(0))] × 100,

where C(t) is the percent of net cell growth over time, Number of Treated Cells(t) is the count of cells treated with drug at each time point, Number of Treated Cells(0) is the count cells treated with drug at time 0 h, Number of Untreated Cells(t) is the count of untreated (control) cells at each time point, Number of Untreated Cells(0) is the count of untreated (control) cells at time 0 h. 

### 2.5. Cell Migration—Wound Healing Assay

The effect of compounds on cell migration was studied using wound healing assay (or scratch assay). Triple-negative breast cancer MDA-MB-231 cells were seeded in 24-well microplate at the cell density 12.5 × 10^4^ cells/cm^2^ (1 mL final volume) and left 24 h to attach and create a confluent monolayer. The cell monolayer was scratched with 200 µL pipette tip, producing a vertical straight wound in the middle of the well; growth medium was removed and wells were washed twice with growth medium to remove all detached cells. Growth medium containing Pd_2_Spm (12.5–200 µM) or cisplatin (6.25–200 µM) was added to the wells, and microplates were imaged using LionheartFX automated microscope at 0, 3, 18, 20, 24 and 48 h. Wound closure was analyzed as a confluence of the initial cell-free area covered by migrating cells at the indicated time points measured with Gen 5 Image Analysis software. Confluence was calculated using the following formula:

C(t) = [(Object Sum Area(t) − Object Sum Area(0))/(I(A) − Object Sum Area(0))] × 100,

where C(t) is the percent wound confluence over time, Object Sum Area(t) is the area covered by cells at each time point, Object Sum Area(0) is the area covered by cells at time 0 h and I(A) is the total area of the 4X image. The area under the curve (AUC) of kinetic time–confluence profiles obtained within 0–18 h for each concentration was calculated (AUC_0–18h_), plotted against the drug concentration and analyzed with nonlinear regression to obtain the half maximal effective concentration (EC50) for Pd_2_Spm and cisplatin.

### 2.6. Quantitative Analysis of Vascular Endothelial Growth Factor (VEGF) Production

MDA-MB-231 cells were seeded in 96-well microplates at the cell density 1.5 × 10^4^ cells/cm^2^ (final volume 200 µL/well) and left 24 h to attach. The growth medium was replaced with growth medium containing Pd_2_Spm (5–20 µM) or cisplatin (5–20 µM), and cells were incubated for 12 h. VEGF concentration in cell culture medium was determined in undiluted samples in duplicate using the Human VEGF Quantikine ELISA Kit (R&D Systems Inc., Minneapolis, MN, USA) according to the manufacturer’s protocol. In brief, 200 μL of conditioned media was added to a 96-well microplate pre-coated with monoclonal antibody for human VEGF and the plate was incubated for 2 h. After washing with wash buffer, enzyme-linked polyclonal antibody specific for human VEGF was added, followed by incubation for 2 h. The plate was washed to remove unbound antibody-enzyme reagent, substrate solution was added and incubated for 30 min and the reaction was terminated by the addition of the stop solution, producing a yellow solution. The optical density at 450 nm with wavelength correction at 540 nm was measured using a Synergy HT microplate reader (BioTek, Winooski, VT, USA). The concentration of VEGF was interpolated from the standard curve (15.6–1000 pg/mL) prepared in parallel.

### 2.7. Mice

Female CBA nude (N:NIH(S)II-nu/nu) mice (6–7 weeks old, 21 animals in total) with combined immunodeficiency [23] were purchased from i3S Animal Facility (Porto, Portugal). Animals were acclimatized at ICBAS-UP Rodent Animal House Facility (Porto, Portugal) at least one week prior to the experiments, were randomly distributed into groups of three per individually ventilated cage and were housed under controlled specific-pathogen-free (SPF) environmental conditions (temperature 22.5 ± 1.5 °C; relative humidity 50 ± 10%; 12 h light/dark cycle) with ad libitum access to water and standard pellet food (4RF21, Mucedola, Italy). Environmental enrichment included corncob bedding, paper roll tube and one large sheet of tissue paper for nesting. Animals were monitored daily for health status and welfare. 

### 2.8. Subcutaneous In Vivo Breast Cancer Xenograft Study

Mice were subcutaneously implanted in the left flank with breast cancer MDA-MB-231 cells (25G needle, 5 × 10^6^ cells in 150 µL of PBS). At day 25 post-implantation, when tumors reached a mean volume of ~250 mm^3^, mice were randomly allocated into three groups (7 animals per group) using computer-generated randomization sequence followed by random group allocation to the treatment with either (A) Pd_2_Spm, (B) cisplatin or (C) vehicle. The Pd_2_Spm (5 mg/kg/day), cisplatin (2 mg/kg/day) or vehicle (PBS + 0.5% DMSO) were administered via intraperitoneal injection (500 µL injection volume) during five consecutive days in the A) Pd_2_Spm, B) cisplatin and C) vehicle group, respectively. The animals were monitored daily for physical activity, wellbeing and measurements of body weight. Tumor measurements were performed by two independent researchers using a digital caliper in two perpendicular diameters of the implant. Tumor volumes were calculated using the Carlsson formula [24]: tumor volume (mm^3^) = 0.5 × largest diameter (mm) × smallest diameter^2^ (mm). Researchers were blinded to treatment allocation while performing outcome measurements. At day 39 post-implantation (end of the study), animals were euthanized with isoflurane followed by cardiac puncture for blood collection. Blood was collected into K3EDTA tubes for hematological and biochemical analyses. Brain, heart, lungs, liver, kidneys, spleen, inguinal lymphatic nodes and tumor were excised, washed in PBS and weighed. Two animals from the vehicle group developed ulcerated tumors during the treatment period (day 28 post-implantation); thus, these animals were euthanized and excluded from the study.

### 2.9. Histological Analysis and Immunohistochemistry

Animal tissues were fixed in 10% buffered formalin, routinely processed and embedded in paraffin wax. Serial sections with 2 μm thickness were cut from each paraffin block; one section was stained with hematoxylin–eosin for histological examination, and the others were used for immunohistochemistry. Histological grading of the mammary carcinomas was performed according to the Nottingham histological grading method [25] based on a semi-quantitative assessment of three histological parameters: tubular formation, nuclear pleomorphism and mitotic figures count. The score of these parameters is used to assign the tumor grade: grade I (well-differentiated tumors), grade II (moderately differentiated tumors) or III (poorly differentiated tumors). 

For the immunohistochemical study, tissue sections were dewaxed and rehydrated. Slides were submitted to proteolytic digestion by immersion in 10% retrieval solution (Dako) and kept in a water bath at 100 °C for 20 min. After blocking of non-specific staining, slides were incubated with primary antibody for Ki-67 (1:50 dilution, clone MIB-1, Dako) overnight at 4 °C. Slides were then stained with Novolink Polymer Detection Systems (Leica Biosystems Inc., Buffalo Grove, IL, USA) following manufacturer’s instructions, counterstained with hematoxylin and permanently mounted. The nuclear Ki-67 staining was analyzed using Fiji’s high-throughput automatic quantitation with Andy’s Algorithm pipeline, as described elsewhere [26]. The immunoreactivity was evaluated, counting at least 600 nuclei/field from 5 representative fields of the lesion at high magnification, avoiding necrotic areas. The ratio of Ki-67 positive nuclei per all counted nuclei was expressed as a percentage (Ki-67 proliferation index).

### 2.10. Detection of Apoptosis with Labeling of DNA Strand Breaks (TUNEL Assay)

The paraffin-embedded tumor sections were deparaffinized, rehydrated through a graded series of ethanol and double distilled water and then stained with In Situ Cell Death Detection Kit (Roche Diagnostics GmbH, Mannheim, Germany) as per manufacturer’s instructions. Apoptosis was quantified at single-cell level by counting the number of cells positive for terminal deoxynucleotidyl transferase (TdT) dUTP nick-end labeling (TUNEL-positive cells). Briefly, tumor sections were incubated with 20 µg/mL proteinase K solution (in 10 mM Tris/HCl, pH 7.4–8) for 30 min at 37 °C. Slides were washed and stained with TUNEL reaction mixture (mixture of terminal deoxynucleotidyl transferase with labeled nucleotides) for 60 min at 37 °C in the dark followed by three washes. Slides were mounted with VECTASHIELD Antifade Mounting Medium with DAPI, and coverslips were sealed. At least twenty nonoverlapping high-power microscope fields of each tumor section were captured while avoiding areas with central necrosis. Fluorescence images were acquired using the LionHeart automated microscope at Ex: 520–560 nm and Em: 570–620 nm.

### 2.11. Biochemical and Hematological Analyses

Red blood cells (RBC), hemoglobin (Hb), hematocrit (Hct), mean corpuscular volume (MCV) and mean corpuscular hemoglobin (MCH) were analyzed in a small aliquot of whole blood using ABX Micros 60 automated analyzer (Horiba ABX SAS, Montpellier, France). The remaining blood was centrifuged at 900× *g* for 15 min, and plasma was separated into aliquots and frozen at −80 °C for biochemical assays. Plasma parameters were analyzed using commercial kits for aspartate aminotransferase—AST (AST CP, A11A01629), alanine transaminase—ALT (ALT CP, A11A01627), cholesterol (CHOL CP, A11A01634), creatinine (Creatinine 120 CP, A11A01868), glucose (Glucose PAP CP, A11A01668), total protein (Total protein CP, A11A01669), triglycerides (Triglycerides CP, A11A01640) and urea (Urea CP, A11A01641). All parameters were determined with an automated analyzer Pentra 400 (ABX Horiba diagnostics, Montpellier, France).

### 2.12. In Vivo CAM Assay

The chicken embryo chorioallantoic membrane (CAM) assay was used to study the effect of tested drugs on neovascularization, as described elsewhere [27]. Briefly, fertilized chicken eggs were incubated at 37.5 °C in a humidified atmosphere with agitation. After 3 days of incubation, 2.5 mL of albumen was removed to detach developing CAM from the shell. A window in the eggshell was cut off to expose the embryo, and then, the eggshell was sealed with paraffin. Incubation of fertilized eggs continued until day 9, when the windows were unsealed and four paper disks previously sterilized and pre-treated with hydrocortisone (a cyclooxygenase inhibitor to avoid inflammatory responses) were placed in direct contact with CAM in each egg (3 PBS-soaked disks and 1 VEGF-soaked (10 ng/mL) disk), and windows were re-sealed with paraffin. On day 11, two of the PBS-soaked disks were treated with Pd_2_Spm (2 to 8 µM) or cisplatin (2 to 8 µM). The remaining two disks were used as negative/vehicle (treated with PBS) and positive (treated with VEGF) controls. The eggs were further incubated two more days, and at day 13, the disks bound to the CAM were removed, placed in PBS and imaged using a contrast-phase microscope (Motic^®^ AE200 inverted microscope, Spectra Services, VWR International) (with a 4X magnification) coupled to a Moticam 5 digital camera. Images were analyzed using the Angiogenesis Analyzer for Fiji [28].

### 2.13. Ethical Considerations 

Handling and care of animals were conducted according to Portuguese (Decreto-Lei n.°113/2013) and European legislation (Directive 2010/63/EU) on the protection of animals used for scientific purposes and were in agreement with the recommendations in the Guide for Care and Use of Laboratory Animals of the National Institutes of Health (NIH). The study protocol was approved by the Ethics Committee for Animal Experimentation of the Faculty of Pharmacy of the University of Porto, Porto, Portugal (permit number 25-10-2015) and by the Ethics Committee of the local animal welfare body (ICBAS-UP ORBEA, Porto, Portugal) with permit number 134/2015. The ARRIVE Guidelines were followed for reporting in vivo experiments [29].

### 2.14. Statistical Data Analysis

Data are expressed as mean ± standard error of the mean (SEM) or medians with corresponding quartiles. Statistical analysis was performed using two-tailed Student’s *t*-test or one-way ANOVA followed by Dunnett’s multiple comparisons for analysis of means. Changes over the time were analyzed with two-way ANOVA followed by Tukey’s multiple comparisons test. Medians were compared with nonparametric Kruskal–Wallis test followed by Dunn’s multiple comparisons test. GraphPad Prism 7 Software (San Diego, CA, USA) was used. A *p*-value < 0.05 was considered statistically significant.

## 3. Results

### 3.1. Pd_2_Spm Showed Selective Antiproliferative Activity for MDA-MB-231 Breast Cancer Cells 

**Figure 1:**
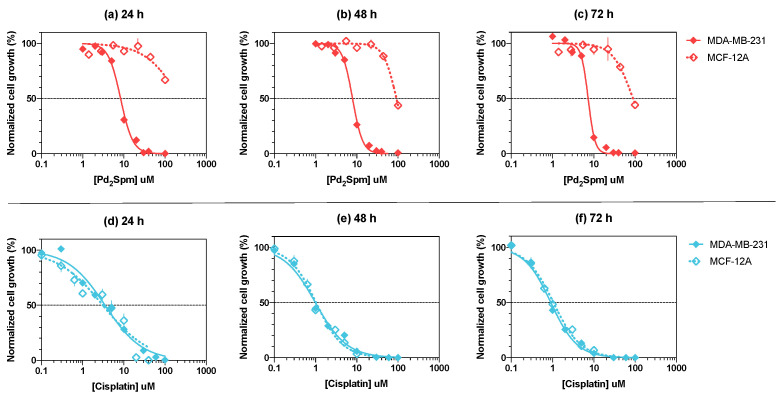
Dose–response curves of Pd_2_Spm (red, **a**–**c**) and cisplatin (blue, **d**–**f**) in breast cancer (MDA-MB-231, solid line) and non-cancer (MCF-12A, dotted line) cells at 24, 48 and 72 h of incubation. Data are expressed as mean ± SEM, *n* = 4. Data points with no visible error bars have errors smaller than the size of the symbol.

The effects of Pd_2_Spm and cisplatin on the proliferation of MDA-MB-231 and MCF-12A cells at 24, 48 and 72 h are shown in Figure 1 as dose–response curves. The full range of antiproliferative activity of Pd_2_Spm and cisplatin on net cell growth (0–100%) was achieved with the tested concentrations (1 to 100 µM). For MCF-12A cells, the highest concentration of Pd_2_Spm (100 µM) did not produce a maximum growth inhibition when compared to the same dosage of cisplatin. A clear separation of dose–response curves obtained for MDA-MB-231 and MCF-12A cells was observed for Pd_2_Spm at 24, 48 and 72 h incubation periods. For cisplatin, an overlap between the dose–response curves for MDA-MB-231 and MCF-12A was detected at these time points. Nonlinear regression analysis of dose–response curves was used to compute the half maximal inhibitory concentration (IC_50_) for Pd_2_Spm and cisplatin (Table 1). For Pd_2_Spm, the IC_50_ values obtained in MDA-MB-231 cells were significantly lower than the IC_50_ values determined in MCF-12A cells, suggesting lower sensitivity of non-cancerous MCF-12A to Pd_2_Spm. For cisplatin, on the other hand, the IC_50_ obtained for MDA-MB-231 and MCF-12A cells did not show significant differences, which is indicative of non-discriminatory antiproliferative effects elicited by cisplatin.

### 3.2. Pd_2_Spm Inhibited In Vitro Migration of MDA-MB-231 Cells

**Figure 2:**
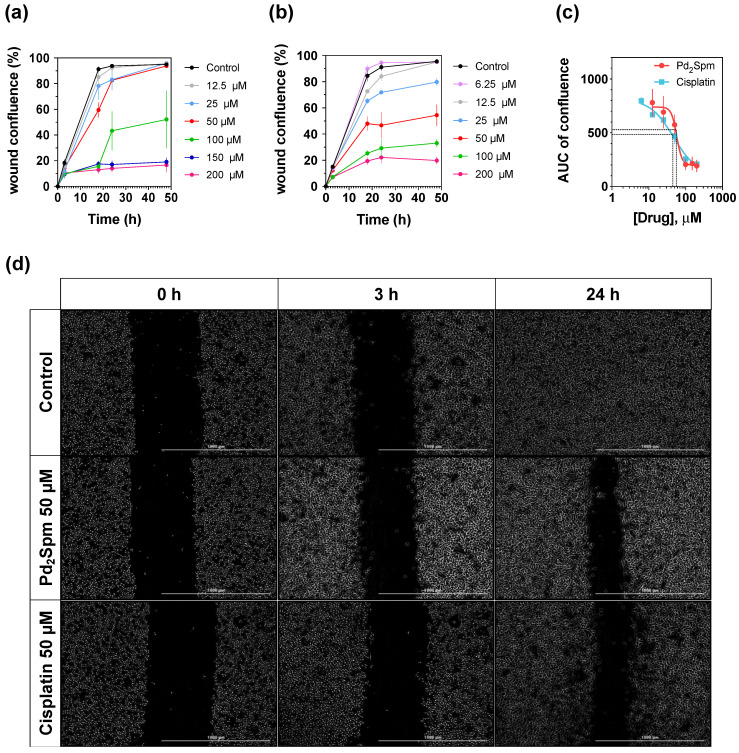
Effects of Pd_2_Spm and cisplatin on migration of MDA-MB-231 cells using scratch/wound healing assay. (**a**) Dose-dependent effect of Pd_2_Spm on migration of MDA-MB-231 cells expressed as wound confluence for tested Pd_2_Spm concentrations. (**b**) Dose-dependent effect of cisplatin on migration of MDA-MB-231 cells expressed as wound confluence for tested cisplatin concentrations. (**c**) Dose–response curve obtained from measuring the area under confluence curve (AUC) between 0 and 18 h of exposure to Pd_2_Spm or cisplatin. (**d**) Representative images from 5 independent experiments showing effects of 0 µM (control) and 50 µM of Pd_2_Spm or cisplatin on cell migration at 0, 3 and 24 h. Scale bar = 1000 µm. Data are expressed as mean ± SEM, *n* = 5. Data points with no visible error bars have errors smaller than the size of the symbol.

The effects of Pd_2_Spm and cisplatin on the migration of MDA-MB-231 cells is depicted in Figure 2. The kinetic profiles (between 0 and 48 h) of migrating cells exposed to different concentrations of either Pd_2_Spm (Figure 2a) or cisplatin (Figure 2b) showed similar dose-dependent effects on cancer cell migration, expressed as a percentage of wound confluency. Untreated cells displayed an almost complete wound closure, reaching a maximum ~100% wound confluence between 18 and 24 h after creation of the scratch in the cell monolayer (Figure 2d). Thus, the area under the curve for the wound confluence–time plot between 0 and 18 h (AUC_0–18h_) was analyzed (Figure 2c) to reliably capture the drugs’ effects on cell migration. The half maximal effective concentrations (EC_50_) of Pd_2_Spm and cisplatin are summarized in Table 2, showing no significant difference in the potency of Pd_2_Spm versus cisplatin in inhibiting the migration of MDA-MB-231 cells. 

### 3.3. Pd_2_Spm Administration Inhibited the Growth of MDA-MB-231 Xenografts in Female Nude Mice

**Figure 3:**
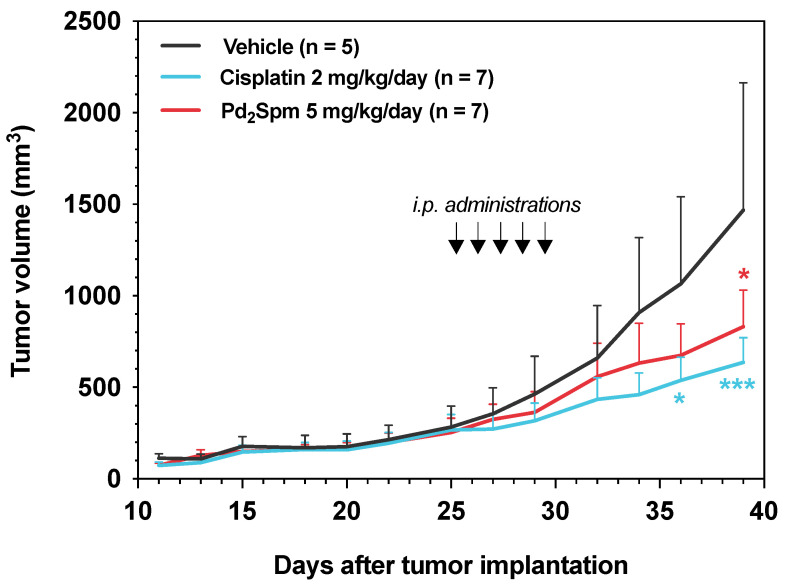
Pd_2_Spm suppresses tumor growth of MDA-MB-231 breast cancer xenografts. Graph of the effect of Pd_2_Spm treatment (5 mg/kg/day), cisplatin treatment (2 mg/kg/day) or vehicle (PBS + 0.5% DMSO) on MDA-MB-231 tumor volume. Drugs were administered intraperitoneally for 5 consecutive days in CBA nude female mice bearing subcutaneously implanted MDA-MB-231 triple-negative human breast cancer tumors. Data are expressed as mean tumor volume (mm^3^) ± SEM and analyzed with two-way ANOVA followed by Tukey’s multiple comparison test. * *p* < 0.05 Pd_2_Spm versus vehicle; *** *p* < 0.001 cisplatin versus vehicle.

To confirm the in vitro findings, we further studied the in vivo effects of intraperitoneal Pd_2_Spm administration on the growth of MDA-MB-231 cancer cells subcutaneously implanted in female nude mice. The cisplatin-treated mice were used as a reference treatment group. The selection of doses of Pd_2_Spm and cisplatin used in vivo took into consideration the doses we used previously for pharmacokinetic and tissue biodistribution studies [15]. The average tumor volumes in mice treated with either Pd_2_Spm (5 mg/kg/day) or cisplatin (2 mg/kg/day) for 5 consecutive days were significantly lower compared to vehicle-treated control mice (*p* < 0.05; Figure 3). At the end of the experiment, the tumor growth reduction (versus the vehicle group) was 43.3% and 56.6% for Pd_2_Spm- and cisplatin-treated animals, respectively. 

### 3.4. Pd_2_Spm Administration Suppressed Cell Proliferation and Induced DNA Damage in Tumors Collected from MDA-MB-231 Breast Tumor-Bearing Mice

**Figure 4:**
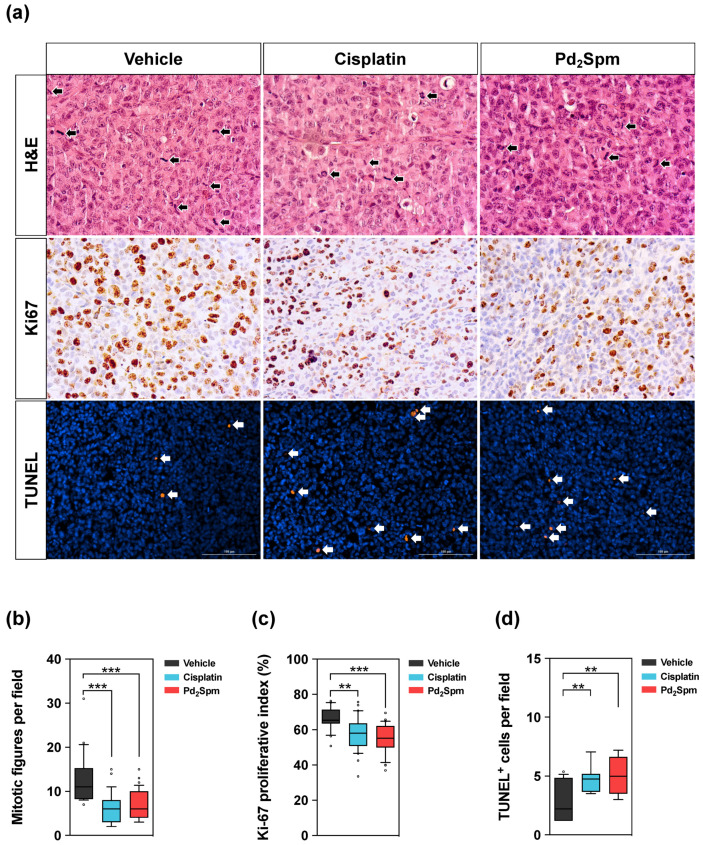
(**a**) Representative micrographs of MDA-MB-231 breast cancer tumors stained with hematoxylin–eosin (H&E, black arrows show mitotic figures), Ki-67 nuclear staining and TUNEL assay staining (white arrows show TUNEL positive cells). (**b**) Quantitative analysis of mitotic figures. (**c**) Quantitative analysis of Ki-67 proliferative index. (**d**) Quantitative analysis of TUNEL-positive cells. Data are shown as box plots with medians analyzed with Kruskal–Wallis test followed by Dunn’s multiple comparisons test. ** *p* < 0.01, *** *p* < 0.001. Five random fields per tumor with at least 600 cells/field were analyzed. Scale bar = 100 µm. Pd_2_Spm 5 mg/kg/day (*n* = 7), cisplatin 2 mg/kg/day (*n* = 7), vehicle (*n* = 5).

All study animals developed malignant neoplasms characterized by a dense population of polyhedral epithelial cells arranged in a solid pattern. Neoplastic lesions exhibited an infiltrative behavior, infiltrating surrounding muscle and adipose tissue, with large central necrotic areas. Neoplastic cells exhibited marked anisocytosis and anisokaryosis, and multinucleated cells and aberrant nuclear shapes were found (Figure 4a—H&E).

Tumors treated with the vehicle showed highly dense cancer cells with atypical nuclei, evidencing intense mitosis. The mitotic count of vehicle-treated animals was significantly increased compared to cisplatin- and Pd_2_Spm-treated groups (Figure 4b). In order to evaluate whether Pd_2_Spm-mediated suppression of tumor growth occurred with a decrease in cell proliferation and/or DNA damage induction, the Ki-67 proliferation index (a well-accepted marker of cellular proliferation, [30]) and the TUNEL assay (marker of DNA damage) were used. Immunoreactivity for Ki-67 in tumor sections from the groups of animals under study (Pd_2_Spm, cisplatin or vehicle) are shown in Figure 4a. The Ki-67 index of tumors was significantly decreased in animals treated with Pd_2_Spm when compared to animals treated with the vehicle. Tumors from cisplatin-treated animals also showed a significant decrease in Ki-67 relative to the vehicle-treated mice (Figure 4c). Analysis of DNA damage showed a significant increase in TUNEL-positive cells in tumors obtained from animals treated with Pd_2_Spm and cisplatin when compared to tumors from vehicle-treated animals (Figure 4d). 

Overall, these results evidenced that Pd_2_Spm administration inhibited the growth of MDA-MB-231 xenografts in vivo by reducing tumor cell proliferation and inducing DNA damage and cell death. Our data also showed that 5-day treatment of tumor-bearing mice with 5 mg/kg/day of Pd_2_Spm or 2 mg/kg/day of cisplatin results in comparable anticancer effects when (i) tumor volume reduction, (ii) tumor proliferative activity decrease (Ki-67 and mitotic count) and (iii) tumor DNA damage increase are compared in Pd_2_Spm- versus cisplatin-treated animals. This may be explained in part by the similarity in the pharmacokinetics of Pd_2_Spm and cisplatin [15], as well as by the similarity in the amounts of administered metals (1.91 mg/kg/day of palladium administered from 5 mg/kg/day of Pd_2_Spm, and 1.3 mg/kg/day of platinum administered from 2 mg/kg/day of cisplatin). 

### 3.5. Impact of Pd_2_Spm on the Weight of MDA-MB-231 Breast Tumor-Bearing Mice

**Figure 5:**
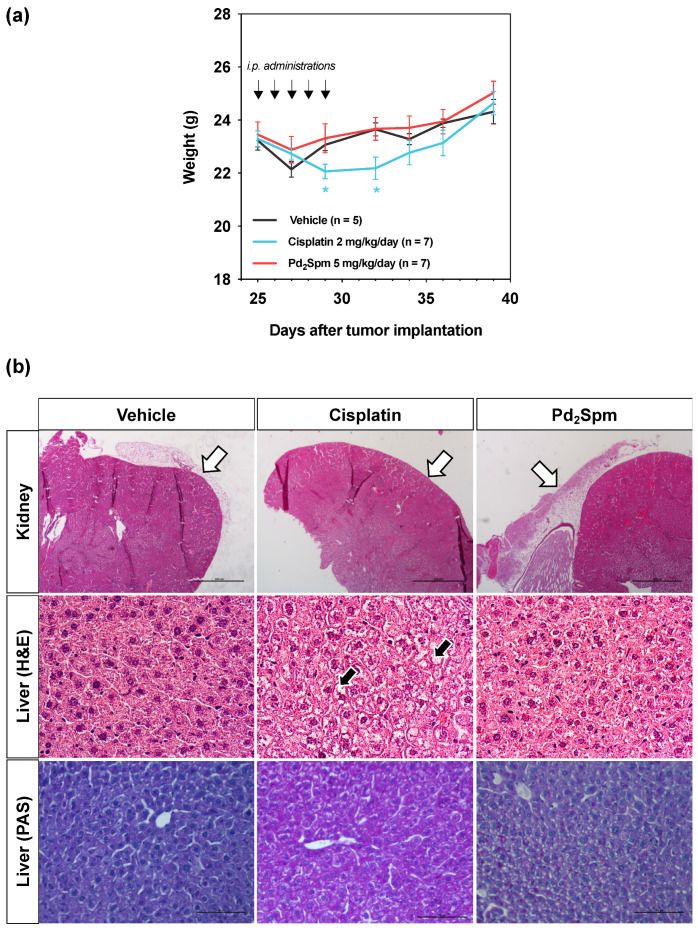
Impact of Pd_2_Spm, cisplatin or vehicle treatment on the body weight of MDA-MB-231 breast tumor-bearing mice. (**a**) Body weight changes of tumor-bearing mice treated intraperitoneally with either Pd_2_Spm (5 mg/kg/day), cisplatin (2 mg/kg/day) or vehicle (PBS + 0.5% DMSO) for 5 consecutive days. Data are expressed as mean weight (g) ± SEM. Two-way ANOVA followed by Tukey’s multiple comparisons test was used to analyze weight change in groups over the time, * *p* < 0.05 cisplatin versus vehicle. (**b**) Representative microphotographs of kidney and liver from animals treated with Pd_2_Spm, cisplatin or vehicle, showing absence of perirenal adipose tissues (white arrows) in cisplatin-treated animals. Hepatocytes of cisplatin-treated animals showing marked cytoplasmic vacuolization and hepatocellular glycogen deposits (black arrows). Scale bar (kidney) = 1000 µm. Scale bar (liver) = 100 µm.

As body weight can be considered a marker of general systemic toxicity, we performed a daily monitoring of the mice’s weight in all groups. A five-day treatment administration induced notable changes in mice’s body weight (Figure 5a): during the initial three days of the treatment, a body weight loss was observed in all study groups, and this trend continued in cisplatin-treated animals also after the administration period. A progressive recovery of the body weight was observed in all groups, and at the end of the experiment, the body weight of all animals was higher compared to the starting value. The cisplatin group demonstrated a slower body weight recovery compared to Pd_2_Spm- and vehicle-treated groups. There was no significant difference in body weight between groups at the end of the procedure. 

In addition to body weight decrease, cisplatin-treated animals showed signs of general toxicity, such as decreased movement/activity, transient dehydration and diarrhea. In turn, no such adverse effects were observed in animals treated with either Pd_2_Spm or the vehicle.

The treatment of animals with Pd_2_Spm, cisplatin or vehicle did not impact the relative weight of the liver, heart, lungs, spleen or brain, with no significant differences being detected among the studied groups (Table 3). On the other hand, the relative weight of the kidneys in cisplatin-treated animals was lower compared to animals treated with the vehicle or Pd_2_Spm. Histopathological evaluation showed a decreased perirenal adipose tissue in cisplatin-treated compared to vehicle-treated animals, which may further support a reduction in the relative weight of the kidneys in cisplatin-treated animals (Figure 5b). No other histopathological changes were observed during histological analysis of the kidneys (data not shown). Regarding the liver, histopathological analysis allowed us to conclude that cisplatin-treated animals exhibited a swollen, pale and finely vacuolated cytoplasm due to intracellular accumulation of glycogen (Figure 5b), as confirmed by periodic acid–Schiff (PAS). This glycogen accumulation was not observed in the livers of vehicle- and Pd_2_Spm-treated animals.

### 3.6. Impact of Pd_2_Spm on the Hematologic and Biochemical Parameters of MDA-MB-231 Breast Tumor-Bearing Mice

**Figure 6:**
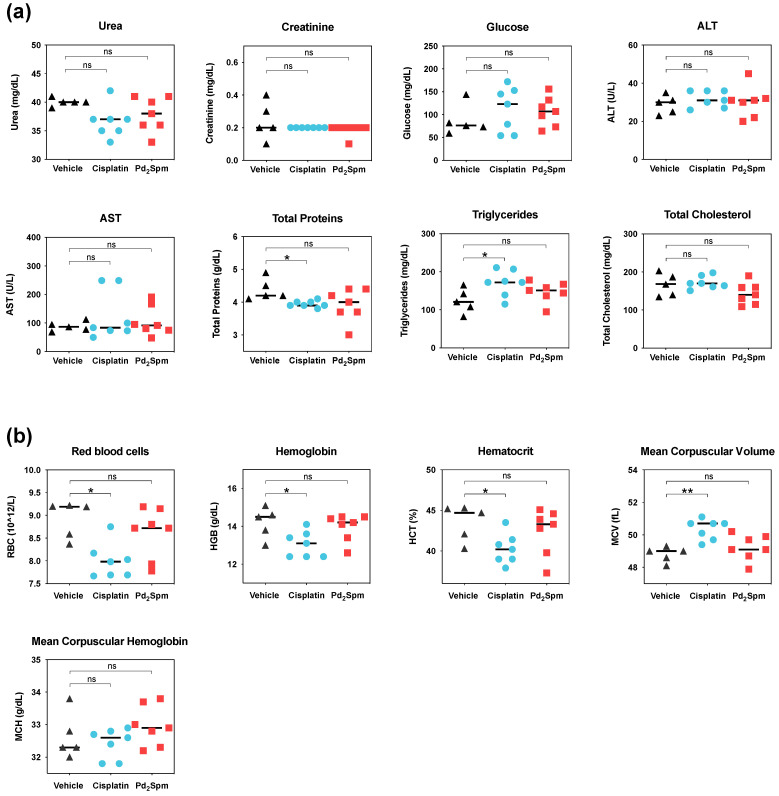
(**a**) Hematologic analysis and (**b**) Biochemical analysis of CBA nude female mice bearing subcutaneously implanted MDA-MB-231 triple-negative human breast cancer tumors treated with either Pd_2_Spm (5 mg/kg/day), cisplatin (2 mg/kg/day) or vehicle (PBS + 0.5% DMSO). Data are shown as individual values with medians, analyzed with Kruskal–Wallis test followed by Dunn’s multiple comparisons test. * *p* < 0.05, ** *p* < 0.01, ns = not significant. Pd_2_Spm (*n* = 7), cisplatin (*n* = 5), vehicle (*n* = 5).

Hematologic analysis (RBC, Hb, Htc, MCV, MCH) of animals treated with Pd_2_Spm revealed no significant deviations from the animals treated with the vehicle (Figure 6a). Cisplatin-treated animals, in turn, showed a decrease in red blood cells, hemoglobin and hematocrit, accompanied by an increase in the mean corpuscular volume, as compared to vehicle-treated animals. Plasma biochemical analysis (urea, creatinine, glucose, AST, ALT, total proteins, triglycerides and total cholesterol) in Pd_2_Spm-treated animals did not reveal changes relative to vehicle-treated animals (Figure 6b). For cisplatin treatment, a decrease in total proteins and an increase in triglycerides were observed as compared to the vehicle-treated group.

### 3.7. Pd_2_Spm Suppressed Neovascularization in Chorioallantoic Membrane and Reduced VEGF Production in MDA-MB-231 Cells

**Figure 7:**
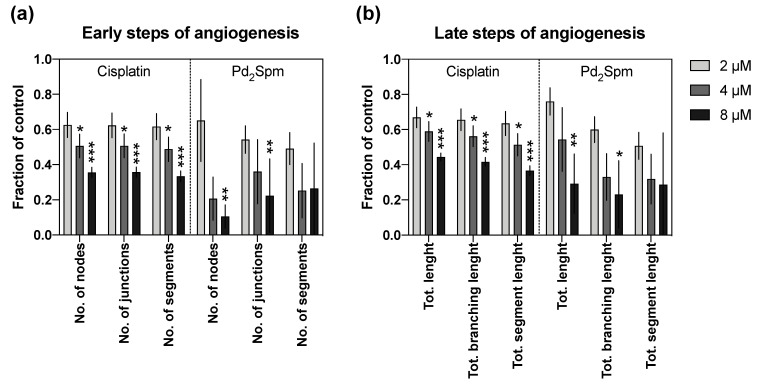
Quantitative results of CAM angiogenesis in the presence of increasing concentrations (2–8 µM) of either cisplatin or Pd_2_Spm. (**a**) Parameters of early steps of angiogenesis: number of nodes, junctions and segments. (**b**) Parameters of later steps of angiogenesis: total length, branching length and segment length of vessels. The results are expressed as means of the fraction of the control ± SEM from 2 independent experiments (*n* = 2, 8 replicates). Significant differences from the vehicle control: * *p* < 0.05, ** *p* < 0.01, *** *p* < 0.001 (one-way ANOVA followed by Dunnett’s multiple comparison test).

As angiogenesis plays an important role in the carcinogenesis of TNBC [31], the antiangiogenic potential of Pd_2_Spm was investigated and compared to cisplatin. We determined the effect of Pd_2_Spm treatment on the neovascularization steps using the CAM assay. Increasing concentrations of Pd_2_Spm (2 to 8 µM) and of cisplatin (2 to 8 µM) were able to decrease, in a dose-dependent manner, the number of nodes, junctions and segments, which are parameters associated with the early steps of angiogenesis (Figure 7a). Similarly, Pd_2_Spm and cisplatin, in the concentrations tested, reduced, in a dose-dependent manner, the total length, total branching length and total segment length, which are linked to the expansion of newly formed vessels in later steps of angiogenesis (Figure 7b). Thus, these data indicate that both Pd_2_Spm and cisplatin have the ability to inhibit angiogenesis, in both the initial and later steps, with a similar potency (*p* > 0.05). 

**Figure 8:**
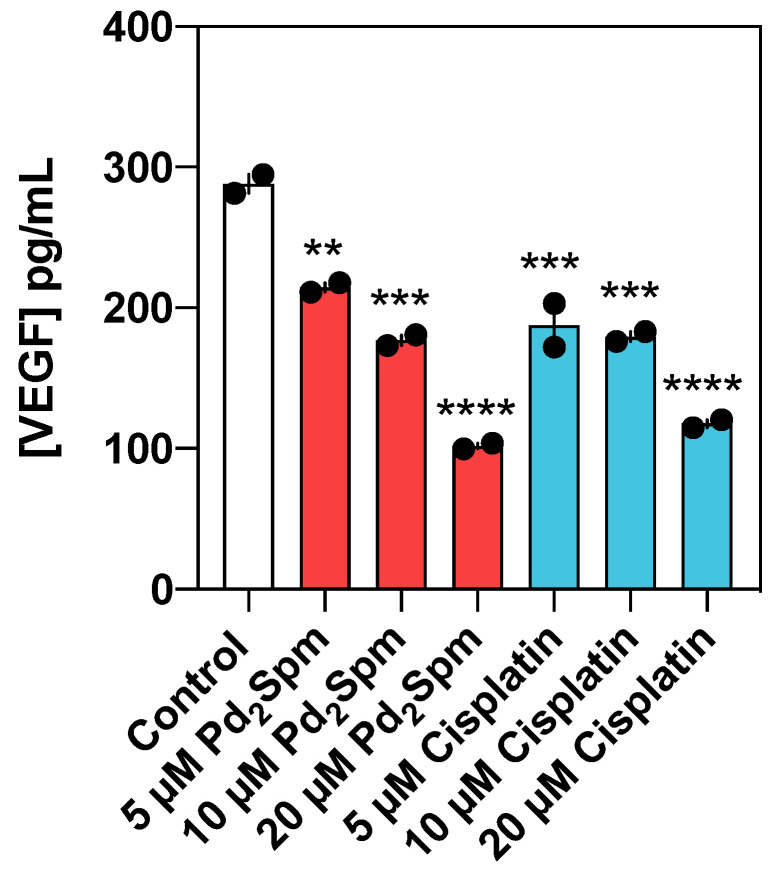
Quantitative results of VEGF secretion by MDA-MB-231 incubated for 12 h with Pd_2_Spm (red) or cisplatin (blue). Data are shown as means ± SEM (*n* = 2) and analyzed with one-way ANOVA followed by Dunnett’s multiple comparison test. Significant differences from the control: ** *p* < 0.01, *** *p* < 0.001, **** *p* < 0.0001.

Since VEGF plays a crucial role in angiogenesis, we have explored the hypothesis that Pd_2_Spm may impact the secretion of VEGF by MDA-MB-231 cells. As shown in Figure 8, both Pd_2_Spm (5–20 µM) and cisplatin (5–20 µM) inhibited VEGF secretion in cell culture medium in a dose-dependent manner. Compared to cisplatin, Pd_2_Spm incubation resulted in a similar decrease in VEGF secretion by MDA-MB-231. 

Together these results indicated that Pd_2_Spm may possess anti-angiogenic potential to be further explored and that this effect can be ascribed, at least in part, to its ability to reduce the secretion of VEGF. 

## 4. Discussion

The in vivo anticancer effects of Pd_2_Spm treatment in mice were studied for the first time, unraveling the promising potential of this dinuclear Pd(II)-polyamine complex regarding its selectivity, efficacy and tolerability as compared to the clinically used Pt(II) drug cisplatin.

The treatment of mice bearing TNBC tumors with 5 mg/kg/day of Pd_2_Spm for five days (cumulative dose of 25 mg/kg) induced significant effects: (i) decrease in tumor volume to a similar extent to that observed in animals treated with 2 mg/kg/day of cisplatin (cumulative dose of 10 mg/kg); (ii) reduction in the number of mitoses in the tumor; (iii) decrease in the proliferative index measured with immunohistochemical marker Ki67; and (iv) increase in TUNEL-positive cells (in the tumor), which are indicative of increased DNA damage. The anti-proliferative and selective effects of Pd_2_Spm towards TNBC cells was evidenced by the significantly higher IC_50_ values obtained for non-cancerous MCF-12A cells (89.5–228.9 µM) as compared to MDA-MB-231 cancer cells (7.3–8.3 µM). This ca. 10-fold higher selectivity of Pd_2_Spm contrasts with the unselective activity of cisplatin, since this conventional Pt drug shows an equivalent potency towards both MDA-MB-231 and MCF-12A cells. Cisplatin’s non-selective antiproliferative activity presently observed corroborates previous reports [32] and may indicate that cisplatin’s non-discriminatory effects are responsible for the appearance of off-target toxicity and adverse effects during chemotherapy. For Pd_2_Spm, the established selectivity further confirms previous studies performed in human fibroblasts [9] and is a particularly promising result that may lead to a considerable reduction in adverse events during chemotherapy. Additionally, Pd_2_Spm consistently shows IC_50_ values within a low micromolar range in MDA-MB-231 [9,11] and MDA-MB-468 TNBC cells [33], which is a requirement for the successful course towards pre-clinical development and further translation into the clinics. Altogether, these in vitro studies sustain the evidence that TNBC is expected to present greater susceptibility to Pd_2_Spm than non-TNBC phenotypes, though the molecular mechanisms for these differential effects are yet to be unraveled. 

The selectivity and higher tolerability of Pd_2_Spm treatment relative to cisplatin was also established from the analysis of the animal welfare, body weight, relative organ weight, histopathological features, hematology and serum biochemistry parameters. Animals treated with Pd_2_Spm did not show changes in body weight, gastrointestinal reactivity (diarrhea) or dehydration, as opposed to animals treated with cisplatin. Moreover, Pd_2_Spm administration did not result in significant variations regarding hematological and biochemical parameters when compared to cisplatin-treated animals which evidenced signs of macrocytic anemia (decrease in red blood cells, hemoglobin and hematocrit together with an increase in mean corpuscular volume). The cisplatin-induced changes in body weight, diarrhea and hematotoxicity were previously described [34,35] and are generally considered as surrogates for the drug’s systemic toxicity. Furthermore, these results support the reduced off-target effects and low toxicity elicited by Pd_2_Spm treatment compared to cisplatin. Interestingly, besides a decrease in the total serum proteins and an increase in serum triacylglycerides, no other parameters related to renal or hepatic functions were altered in cisplatin-treated animals. In contrast, Pd_2_Spm treatment did not trigger any of these changes.

Since angiogenesis and cell migration are significant contributors to TNBC pathogenesis, particularly regarding its metastatic potential and aggressive biology, the development of agents able to suppress cancer cell proliferation, migration and angiogenesis are highly desired. In this study, Pd_2_Spm-mediated effects against cancer cell invasion yielded promising results since Pd_2_Spm: (i) inhibited the migration of MDA-MB-231 cells; (ii) inhibited angiogenesis in CAM; and (iii) suppressed VEGF secretion by MDA-MB-231 cells with similar potency to cisplatin. These results are in line with previous reports that revealed that cisplatin’s anti-migratory and antiangiogenic effects, although the underlying mechanism(s) are still largely unknown [36,37]. Regarding the effects of Pd(II) compounds, there is limited information in the literature describing their ability to suppress angiogenesis and cell migration. So far, studies with the mononuclear palladium (II) saccharinate complex of terpyridine [PdCl(terpy)](sac)·2H_2_O have shown inhibition of the migration of endothelial cells (HUVEC) in the range of 12.5–50 µM and have also shown inhibition of angiogenesis in CAM assay at a concentration of 5 mg/mL [38]. In the current study, Pd_2_Spm significantly inhibited angiogenesis at an 8 µM concentration (corresponding to 4.46 µg/mL of Pd_2_Spm), impacting both early and late steps of angiogenesis and further supporting the previous reports [11]. Nevertheless, the mechanism behind this activity is still unexplored and requires further study.

The in vivo anticancer activity of Pd(II)-based compounds against TNBC is still largely unexplored, and to date, there are only a few in vivo studies in rodents regarding Ehrlich ascites carcinoma (EAC), which is an undifferentiated murine mammary adenocarcinoma sensitive to chemotherapy and not classified as TNBC [39,40]. The Pd complex of terpyridine with saccharinate, also known as [Pd(sac)(terpy)](sac)·4H_2_O, administered intraperitoneally at a dose of 2 mg/kg (two injections per week for 2 weeks, up to a cumulative dose of 8 mg/kg) was found to reduce tumor volume by 67.5%, but one mouse died during treatment (1 of 10), indicating the toxicity and poor tolerability of this compound [39]. Another study focused on the similar derivative [PdCl(terpy)](sac)·2H_2_O found that at a dose of 3 mg/kg (two injections per week for 2 weeks, up to a cumulative dose of 12 mg/kg), it reduced tumor volume by 69.8%, and one mouse died during treatment (1 of 9) [40]. Additionally, mice treated with 4 mg/kg of cisplatin (two injections per week for 2 weeks up to a cumulative dose of 16 mg/kg) showed 36.4% tumor volume reduction in a first study with two associated deaths [39] and a in 46.7% tumor volume reduction in a second study with no associated deaths [40]. When compared to the present results, it is clear that Pd_2_Spm shows a good ability to effectively reduce the growth of TNBC tumors by 43.3% at a cumulative dose of 25 mg/kg (5 mg/kg/day of Pd_2_Spm for 5 consecutive days) with no associated deaths, while cisplatin reduced the tumor growth by 56.6% at a cumulative dose of 10 mg/kg (2 mg/kg/day of cisplatin for 5 consecutive days). The selection of the 5 mg/kg/day dose of Pd_2_Spm accounted for Pd_2_Spm’s pharmacokinetics and differential accumulation of palladium in cancer cells that were explored in previous studies [15]. Moreover, the dose selection also reflected the different molar ratios of palladium and platinum atoms in Pd_2_Spm and cisplatin molecules, respectively (i.e., 5 mg/kg/day of Pd_2_Spm corresponded to 1.91 mg/kg/day of the administered dose of palladium, and 2 mg/kg of cisplatin corresponded to 1.3 mg/kg/day of the administered dose of platinum) [15]. Finally, given the fact that the dose of Pd_2_Spm used in this study yielded a promising efficacy and good tolerability, future studies may be focused on a further increase in therapeutic outcomes using different treatment schedules and/or higher Pd_2_Spm concentrations. 

## 5. Conclusions

This study constitutes the first proof-of-concept regarding the in vivo activity of Pd_2_Spm as a potential candidate chemotherapeutic for TNBC treatment. The in vivo administration of Pd_2_Spm in TNBC tumor-bearing mice resulted in tumor growth inhibition to a similar extent to that in animals treated with cisplatin. Furthermore, the systemic toxicity (hematotoxicity, weight loss, diarrhea and biochemical alterations) observed in cisplatin-treated animals was not verified in Pd_2_Spm-treated mice. Here we have provided encouraging evidence of the selective activity of Pd_2_Spm towards TNBC with minimal effects to non-cancerous breast cells, both in vitro and in vivo. Therefore, Pd_2_Spm may become a promising potential Pd(II) drug candidate for the treatment of TNBC.

## Figures and Tables

**Table 1:** The half maximal inhibitory concentration (IC_50_) of Pd_2_Spm and cisplatin in triple-negative human breast cancer (MDA-MB-231) and non-cancer breast (MCF-12A) cells at 24, 48 and 72 h of incubation.

Drug	Time	MDA-MB-231		MCF-12A		*p*-Value ^a^
		IC_50_ in µM [95% CI]	logIC_50_ ± SEM	IC_50_ in µM [95% CI]	logIC_50_ ± SEM	
Pd_2_Spm	24 h	8.3 [7.4–9.3]	−5.081 ± 0.024	228.9 [107.9–114412]	−3.640 ± 0.215	0.0006
	48 h	7.9 [7.1–8.8]	−5.105 ± 0.022	91.5 [81.5–104.3]	−4.039 ± 0.023	<0.0001
	72 h	7.3 [6.6–8.0]	−5.137 ± 0.021	89.5 [66.4–159.0]	−4.048 ± 0.068	<0.0001
Cisplatin	24 h	3.5 [2.9–4.1]	−5.459 ± 0.036	3.1 [2.3–4.1]	−5.510 ± 0.062	0.5308
	48 h	1.0 [0.9–1.1]	−6.005 ± 0.027	1.0 [0.9–1.1]	−5.988 ± 0.029	0.6954
	72 h	0.9 [0.8–1.0]	−6.043 ± 0.020	1.0 [0.9–1.2]	−5.981 ± 0.026	0.1176

IC_50_ values are expressed in µM with corresponding 95% confidence intervals and as mean logIC_50_ ± SEM, *n* = 4; ^a^ Student’s *t*-test comparison of logIC_50_ means between the two cell lines at a specified time.

**Table 2:** Migration of MDA-MB-231 cells (logEC50 values of Pd_2_Spm and cisplatin).

Drug	logEC50	*p*-Value
Pd_2_Spm	−4.243 ± 0.113 (57.2)	0.631
Cisplatin	−4.329 ± 0.130 (46.9)	

Data are expressed as mean logEC_50_ ± SEM (EC_50_ in µM), *n* = 5.

**Table 3:** Relative organ weight after treatment with either Pd_2_Spm, cisplatin or vehicle.

	Relative Organ Weight (%) ^a^		
Organ	Vehicle	Cisplatin 2 mg/kg/day	Pd_2_Spm 5 mg/kg/day
	(*n* = 5)	(*n* = 7)	(*n* = 7)
Liver	6.08 ± 0.55	6.50 ± 0.34	6.14 ± 0.29
Heart	0.53 ± 0.06	0.53 ± 0.06	0.53 ± 0.06
Lungs	0.73 ± 0.11	0.77 ± 0.08	0.77 ± 0.08
Spleen	0.31 ± 0.10	0.33 ± 0.08	0.32 ± 0.11
Brain	1.85 ± 0.10	1.79 ± 0.14	1.79 ± 0.16
Kidneys	1.46 ± 0.05	1.29 ± 0.10 *	1.43 ± 0.10

^a^ The relative organ weight (mean % ± SEM) was calculated as organ weight (g)/body weight (g) × 100%. Data analyzed with one-way ANOVA followed by Dunnett’s multiple comparisons *t*-test. * *p* < 0.05 cisplatin versus vehicle.

## Data Availability

The data presented in this study are available on request from the corresponding author (M.V.).
